# The estimated disease burden of acute COVID-19 in the Netherlands in 2020, in disability-adjusted life-years

**DOI:** 10.1007/s10654-022-00895-0

**Published:** 2022-08-11

**Authors:** Scott A. McDonald, Giske R. Lagerweij, Pieter de Boer, Hester E. de Melker, Roan Pijnacker, Lapo Mughini Gras, Mirjam E. Kretzschmar, Gerco den Hartog, Arianne B. van Gageldonk-Lafeber, RIVM COVID-19 surveillance, epidemiology team, Agnetha Hofhuis, Anne Teirlinck, Alies van Lier, Bronke Boudewijns, Miek de Dreu, Anne-Wil Valk, Femke Jongenotter, Carolien Verstraten, Gert Broekhaar, Guido Willekens, Irene Veldhuijzen, Jan Polman, Jan van de Kassteele, Jeroen Alblas, Janneke van Heereveld, Janneke Heijne, Kirsten Bulsink, Lieke Wielders, Liselotte van Asten, Liz Jenniskens, Loes Soetens, Maarten Mulder, Maarten Schipper, Marit de Lange, Naomi Smorenburg, Nienke Neppelenbroek, Patrick van den Berg, Priscila de Oliveira Bressane Lima, Rolina van Gaalen, Sara Wijburg, Shahabeh Abbas Zadeh Siméon de Bruijn, Senna van Iersel, Stijn Andeweg, Sjoerd Wierenga, Susan Lanooij, Sylvia Keijser, Tara Smit, Don Klinkenberg, Jantien Backer, Pieter de Boer, Scott McDonald, Amber Maxwell, Annabel Niessen, Brechje de Gier, Danytza Berry, Daphne van Wees, Dimphey van Meijeren, Eric R. A. Vos, Frederika Dijkstra, Jeanet Kemmeren, Kylie Ainslie, Marit Middeldorp, Marjolein Kooijman, Mirjam Knol, Timor Faber, Albert Hoek, Eveline Geubbels, Birgit van Benthem, Hester de Melker, Jacco Wallinga, Arianne B. van Gageldonk-Lafeber, Susan Hahné, Susan van den Hof, Susan van den f, Jacco Wallinga

**Affiliations:** 1 Center for Infectious Disease Control, National Institute for Public Health and the Environment (RIVM), Bilthoven, The Netherlands; 2 University Medical Center Utrecht, Utrecht University, Utrecht, The Netherlands; 3 Department of Biomedical Data Sciences, Leiden University Medical Center, Leiden, The Netherlands

**Keywords:** COVID-19, Disability-adjusted life-years, Disease burden, Pandemic, Netherlands

## Abstract

The impact of COVID-19 on population health is recognised as being substantial, yet few studies have attempted to quantify to what extent infection causes mild or moderate symptoms only, requires hospital and/or ICU admission, results in prolonged and chronic illness, or leads to premature death. We aimed to quantify the total disease burden of acute COVID-19 in the Netherlands in 2020 using the disability-adjusted life-years (DALY) measure, and to investigate how burden varies between age-groups and occupations. Using standard methods and diverse data sources (mandatory notifications, population-level seroprevalence, hospital and ICU admissions, registered COVID-19 deaths, and the literature), we estimated years of life lost (YLL), years lived with disability, DALY and DALY per 100,000 population due to COVID-19, excluding post-acute sequelae, stratified by 5-year age-group and occupation category. The total disease burden due to acute COVID-19 was 286,100 (95% CI: 281,700–290,500) DALY, and the per-capita burden was 1640 (95% CI: 1620–1670) DALY/100,000, of which 99.4% consisted of YLL. The per-capita burden increased steeply with age, starting from 60 to 64 years, with relatively little burden estimated for persons under 50 years old. SARS-CoV-2 infection and associated premature mortality was responsible for a considerable direct health burden in the Netherlands, despite extensive public health measures. DALY were much higher than for other high-burden infectious diseases, but lower than estimated for coronary heart disease. These findings are valuable for informing public health decision-makers regarding the expected COVID-19 health burden among population subgroups, and the possible gains from targeted preventative interventions.

**Supplementary Information:**

The online version contains supplementary material available at 10.1007/s10654-022-00895-0.

## Background

As in most other European countries, SARS-CoV-2 infection was first detected in early 2020 in the Netherlands. The COVID-19 epidemic during 2020 and early 2021 was characterised by two waves, the first of which peaked in mid-March then subsided to a very low level by the end of June; the second wave was distinguished by a slow increase during the summer months that transitioned into a steep rise in positive cases from about mid-September, reaching a peak in the last week of December and then dropping to a relative low by the first week of February 2021 [1]. Because testing eligibility and testing capacity for SARS-CoV-2 infection evolved since the start of the epidemic, time-series of hospitalised and fatal cases provides a better picture of the epidemic severity over time compared with notified cases [2, 3] (Fig. S1). The first wave was responsible for a high burden on hospital and intensive care unit (ICU) resources, as well as for mortality, with an estimated 10,000 deaths from COVID-19 occurring between February and June 2020 in a population of approximately 17 million persons [2]. In 2020 alone 20,173 deaths were attributed to COVID-19 [3].

A key component of ongoing infectious disease surveillance activities in the Netherlands is the routine calculation of the annual disease burden for a large set of infectious diseases using the summary measure disability-adjusted life-years (DALY) [4, 5]. In anticipation of the outbreak of SARS-CoV-2 infection in February 2020, COVID-19 was added to the set of statutory notifiable diseases, and collaborative actions were taken to develop a framework for the computation of disease burden due to COVID-19, resulting in a published methodological protocol [6]. This approach involved combining all relevant surveillance data sources to enable the estimation of disease burden, in DALY, due to COVID-19. The DALY measure is useful for assessing, and thus for comparing the population impact of infectious diseases, because mortality and morbidity – both from acute and chronic disease phases, as well as long-term sequelae – are integrated into a single summary value that measures loss of health [7].

Besides the strain on the healthcare system, the COVID-19 epidemic has had a large direct effect on population health. The loss of healthy life years in those affected—whether with self-limiting mild symptoms, or requiring hospital and/or ICU admission, or leading to premature death – is recognised as being substantial [8]. In this study we quantified the direct health burden – in DALY – due to acute COVID-19 in the Netherlands in the 2020 calendar year, and set this burden in context by comparing to the disease burden estimated for other countries in the same time period, and to the burden of other diseases in the Netherlands. Our primary objectives for estimating DALY were to inform public health policy decision-making and to compare the disease burden of acute COVID-19 across countries and to other diseases. This study builds upon earlier, preliminary disease burden estimates produced for the first COVID-19 wave (27 February to 30 June 2020) in the Netherlands [4, 9]. The disease burden experienced by persons in different age-groups or workplace situations may not be proportionally distributed because of differences in the risk of severe disease and variation in exposure risk. Therefore, as a second objective we also estimate disease burden stratified by five-year age-group and occupation category.

## Methods

We defined a clinical pathway progression model for COVID-19, by first assuming the existence of health states representing three degrees of clinical severity: *mild/moderate* (acute symptomatic illness), *severe* (requiring hospitalisation), and *critical* (requiring ICU care) [4, 6], with asymptomatic infection by definition not contributing to disease burden (Fig. S2). Confirmed SARS-CoV-2 positive cases who develop *mild/moderate* symptomatic COVID-19 can progress to *severe* disease and then to the *critical* disease state. Due to insufficient data we do not estimate the disease burden attributable to post-acute long-term sequelae ('long-COVID'). Death due to COVID-19 is assumed possible following any of these three disease states (Table 1, Fig. S2). We estimated disease burden for the period encompassing the date of the first recorded case (27 February 2020) until 31 December 2020. Note that this calendar year period encompasses the first wave (until 30 June 2020) and most of the second wave. For more details on the methodology used for disease burden estimation, see [4, 6, 9].

**Table 1:** Summary of data sources and DALY parameters for analysis period 27 February through 31 December 2020

Parameter/data	Value/description	Source
Conditional life expectancy	For < 1 year, 1–4 years, and then 5-year bins until 95+ years	GBD-2019 [20]
Incidence, Mild/moderate cases	Estimated symptomatic infection cases derived from modelled age-specific seroprevalence (from PICO-3), Osiris notified cases and estimated underascertainment, and adjusted for the estimated age-specific proportion symptomatic	
Disability duration, Mild/moderate	10 days	[40, 41]
Disability weight, Mild/moderate	0.051 'Infectious disease acute episode, moderate'	[42]
Incidence, Severe cases	Cumulative NICE non-ICU hospital admissions, per 5-year age-group. Assumed Poisson distributed	NICE [10]
Underreporting adjustment, Severe	1.10 (95% CI: 1.06–1.18) [Uniform distribution]. Internally calculated based on Osiris and NICE datasets	
Disability duration, Severe	8 days	NICE [43]
Disability weight, Severe	0.133 'Infectious disease acute episode, severe'	[42]
Incidence, Critical cases	Cumulative NICE ICU admissions, per 5-year age-group. Assumed Poisson distributed	NICE [10]
Underreporting adjustment, Critical	1.0	
Disability duration, Critical	19 days. Derived based on NICE data from the first wave only. (NB. a longer preceding Severe phase of 10 days duration is assumed for patients admitted to ICU)	NICE [44]
Disability weight, Critical	0.655 'Intensive care unit admission'	[45]
Deaths	Registered COVID-19 deaths published by Statistics Netherlands (per 5-year age-group)	[3]

### Data sources and other parameters

Because data on the incidence of each clinical severity category was not available from a single data source, we drew upon data from several sources. For non-ICU hospital admissions and ICU admissions (informing the numbers of persons in the *severe* and *critical* health states, respectively), data were provided by National Intensive Care Evaluations (NICE) [10]. As completeness of the data for ICU admissions was deemed to be 100%, correction for underreporting was not necessary; however, adjustment for underestimation of non-ICU hospital admissions was needed (Table 1). All hospitals with an ICU department report their admissions to NICE, but reporting of non-ICU hospital admissions was only initiated during the COVID-19 pandemic, and as a consequence some hospitals did not report non-ICU admissions.

For the number of COVID-19 deaths, we used data on registered COVID-19 deaths, as published by Statistics Netherlands [3].

For the cumulative incidence of *mild/moderate* (symptomatic) infection, we used two principal data sources. The first was the third round of the national-level seroprevalence survey, the PIENTER Corona study (PICO-3), conducted in late September 2020 [12] to estimate the population-level seroprevalence. This source provided age-specific estimates of the cumulative incidence of infection (both symptomatic and asymptomatic) on the basis of IgG serostatus, which was then adjusted by age-specific estimates of the proportion symptomatic [13] (derived using the earlier serosurvey rounds PICO-1 [14] and PICO-2 [15]), where 'symptomatic' was defined according to the ECDC case definition (fever and/or cough and/or shortness of breath and/or loss of smell/taste). The age-aggregated symptomatic proportion using this approach was estimated at 35%, but as this proportion varied by age, age-group specific estimates were applied [13].

The second source informing the cumulative incidence of *mild/moderate* infection consisted of age-group specific notified positive cases reported in Osiris [11]. We then adjusted these data for case ascertainment and the estimated proportion symptomatic (see below). The distribution over a pre-defined set of occupation categories (see below) was also derived from Osiris [11], which contains information regarding occupation for each notified case, and denominator population sizes for each occupation category were obtained from Statistics Netherlands [16].

### Cumulative incidence of symptomatic infection

Estimation of the cumulative incidence of symptomatic infection (SI) in 2020 required a two-step approach. We used PICO-3 age-specific seroprevalence to first estimate the cumulative incidence of infection in the period until the third week of September, and then integrated several data sources to estimate the cumulative incidence of infection from this date until the end of the year.

#### Analysis period 1

For the period from 27 February until 24 September 2020 (which covers the first wave and the early part of the second wave), the cumulative SI incidence was estimated based on age-group specific seroprevalence from the PICO-3 study conducted between 22 September and 12 November 2020 (the 'index' date of 25 September was selected as 90% of participants had responded by 9 October, and then we subtracted 14 days to take the development of an IgG response into consideration). Observed seroprevalence was weighted by sex, age, ethnicity, and urbanisation to match the Netherlands population distribution in 2020, corrected for test performance [15] and seroreversion, and then adjusted for the estimated age-group specific symptomatic proportion. Observed seroprevalence was weighted on a set of sociodemographic characteristics (sex, age, ethnic background, urbanization) to match the population distribution of the Netherlands population in 2020 and corrected for test specifics.

#### Analysis period 2

For this period, defined as 25 September until the end of 2020, we used an alternative approach to estimate cumulative SI incidence. We based this on the number of notified positive cases (in Osiris) in this period, adjusted for underascertainment. We pooled nine estimates of the ascertainment of all infected persons by notified cases based on population-level survey data from England (nine occasions when members of a community cohort underwent virological testing, conducted by the Office for National Statistics (ONS) between 18–24 September and 22–28 November 2020). Using these data entailed making two strong assumptions: (i) testing policy, availability of tests, and willingness to be tested in England are broadly similar to the Netherlands over this period, and (ii) ascertainment does not vary with age. The pooled age-independent ascertainment estimate is 38.7% (95% CI: 36.1–41.4%). We then estimated cumulative infection incidence for the period 25 September through 31 December 2020 by synthesising estimates using this approach (while adjusting the precision of estimated ascertainment for multiple age-groups) with those from a second approach. This approach, for age-groups 30–34 and older only, was based on the observation of a relatively constant ratio between infections and hospital admissions, and involved multiplying age-group specific cumulative hospital admission ratios by the cumulative incidence as of 24 September 2020.

### Estimation of disease burden

We stratify disease burden estimates by age-group and by occupation category, and present both absolute DALY and DALY per 100,000 persons (a measure of relative burden, that adjusts for population size). In addition, we calculated DALY per 1000 infected persons comparing the first and second analysis periods (the first period comprises the first wave plus July, August and most of September; the second period captures the rise in COVID-19 deaths that began near the end of September [1] (Fig. S1)). Thus, these period-specific estimates permit the severity of the epidemic in terms of the disease burden per infection to be roughly compared across waves (although the mortality consequences of the second wave would extend until February 2021).

#### Computation of YLD and YLL

Estimation of the disease burden in DALY incorporates the years of life lost (YLL) due to premature mortality, and years lived with disability (YLD) [7]. YLD is calculated for each non-fatal health state in the clinical pathway progression diagram (Fig. S2) by multiplying the number of persons entering that state by the average duration in the state and the severity (disability weight; scale of 0 to 1 with 0 indicating no disability) [6, 17] (see also Supplementary Materials). YLD is calculated for each of health state separately and then summed to express the total loss of health due to morbidity in the population. All DALY parameter values are provided in Table 1. For more information on the history of and issues concerning the DALY measure, see [7, 18, 19]. We constructed 95% confidence intervals (CIs) for outcome measures using Monte-Carlo simulation; for instance for YLD 95% CIs were derived by sampling from the distributions specifying the incidence and duration parameters.

To estimate YLL, conditional life expectancy values were adopted from the GBD 2019 study [20]. For informing public health policy decision-making, the appropriate counterfactual is an aspirational life-table derived from low mortality risks. Thus, YLL were not adjusted for pre-existing medical conditions, lifestyle risk factors, high risk occupations, receipt of palliative care, frailty, or living circumstances (such as nursing home residents), nor we did not use distinct life-tables for subpopulations defined by these variables, approaches adopted by other researchers [22–23] which are compatible with other objectives, such as health economic evaluations. Our rational is that if YLL is used to guide the deployment of resource-limited prevention and/or treatment initiatives, these subpopulations could be placed at a disadvantage. Thus on grounds of equity, the YLL component of our DALY estimates are dependent only on age at death.

#### DALY stratified by occupation category

**Fig. 1:**
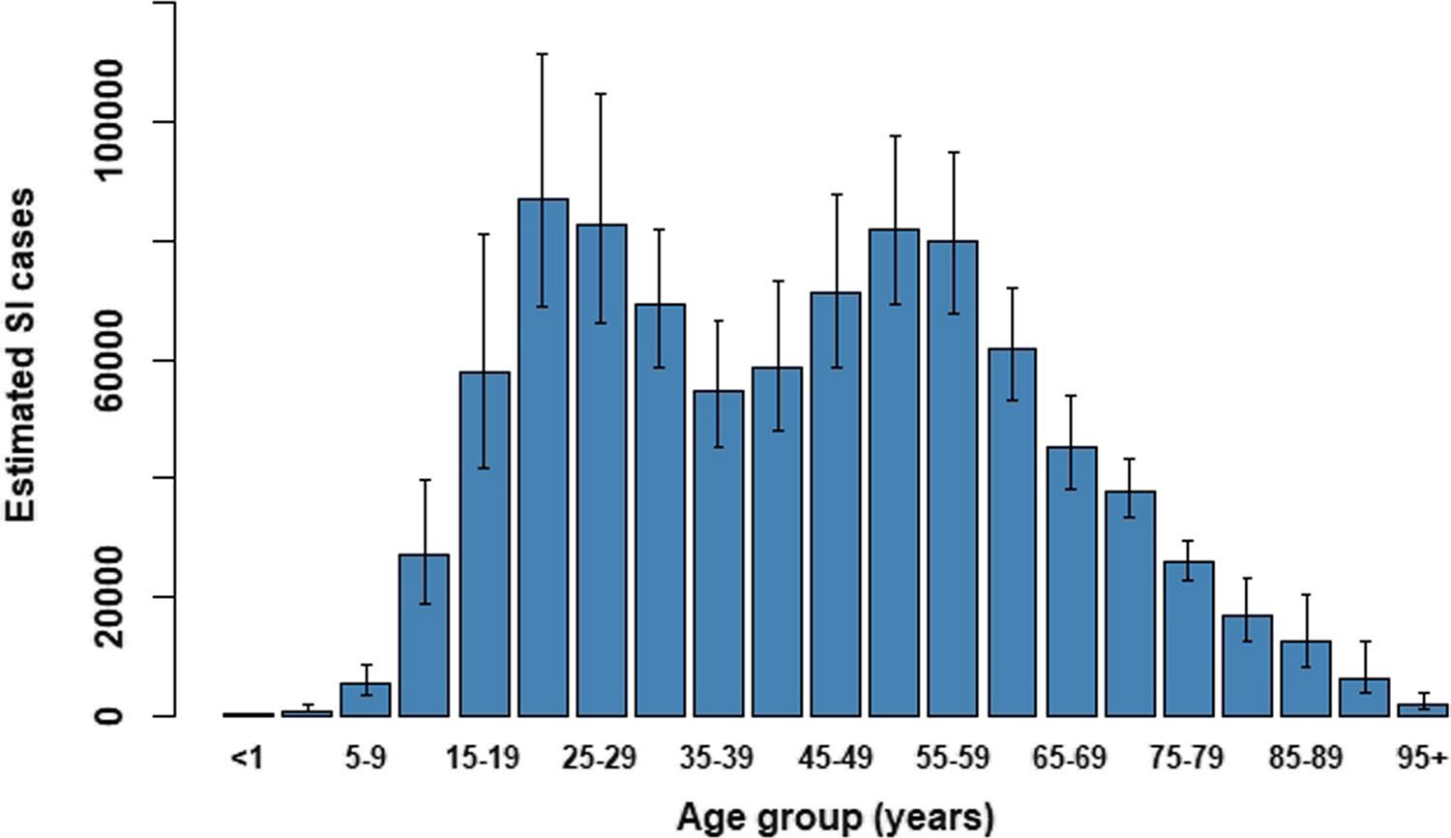
Estimated cumulative incidence of symptomatic infection (SI) per 5-year age-group with 95% CIs, 27 February through 31 December 2020

For the per-capita DALY estimates stratified by occupation category, estimates of the denominator–the total number of persons in each category (from CBS), stratified by age-group–are required. As the available information from Statistics Netherlands [16] contains the number of persons in each occupation per 10-year age-group (15–24, 25–34, … 65–74) only, we needed to map the 10-year denominator age-groups to 5-year age-groups (Fig. 1).

We first defined occupation categories according to notified case data in Osiris (Table S1), and then plotted the distribution over occupation category, stratified by 5-year age-group (Fig. 4). Estimation of the occupation category denominators required the set of occupation categories reported in Osiris to be mapped to the 4 digit code categories used by CBS (see Table S1 for the adopted mapping).

We then applied the distribution over occupation category (inferred from the full analysis period) to both YLD and YLL. Note that deriving a single occupation distribution from the full analysis period conflates impacts from: the evolution of testing policy over time, closures of certain parts of the economy, the various (sector-specific) preventative measures in place, and the periods in which lockdown was imposed. Because a substantial proportion of notifications (14%) had occupation 'Not known', we applied simple univariate imputation to re-distribute the not-known category among the observed occupation categories.

We next mapped the occupation category distribution (determined on the basis of 10-year age-groups) from Osiris to 5-year age-groups used for DALY calculation; e.g., the distribution inferred for 25–34 years was applied to both 25–29 and 30–34 years, and the assumed denominator populations for each of these two 5-year age-groups is the 10-year age-group denominator population weighted according to the national population sizes of the 25–29 and 30–34 years age-groups. Importantly, the occupation distribution is calculated separately within each age-group and applied to the DALY within each age-group. All estimates of DALY per occupation category are restricted to the 'working population' age range (defined as age 20 through 69 years).

In supplementary analysis we compared the per-capita COVID-19 disease burden among individuals aged 80 years or older receiving long-term institutional health care (including those residing in nursing homes, elderly and disabled care residences, but also those receiving full-time homecare), based on COVID-19 deaths and the total number of persons receiving long-term care published by Statistics Netherlands [24, 25], to the burden among community residents in the same age-group.

## Results

### DALY stratified by age-group

**Fig. 2:**
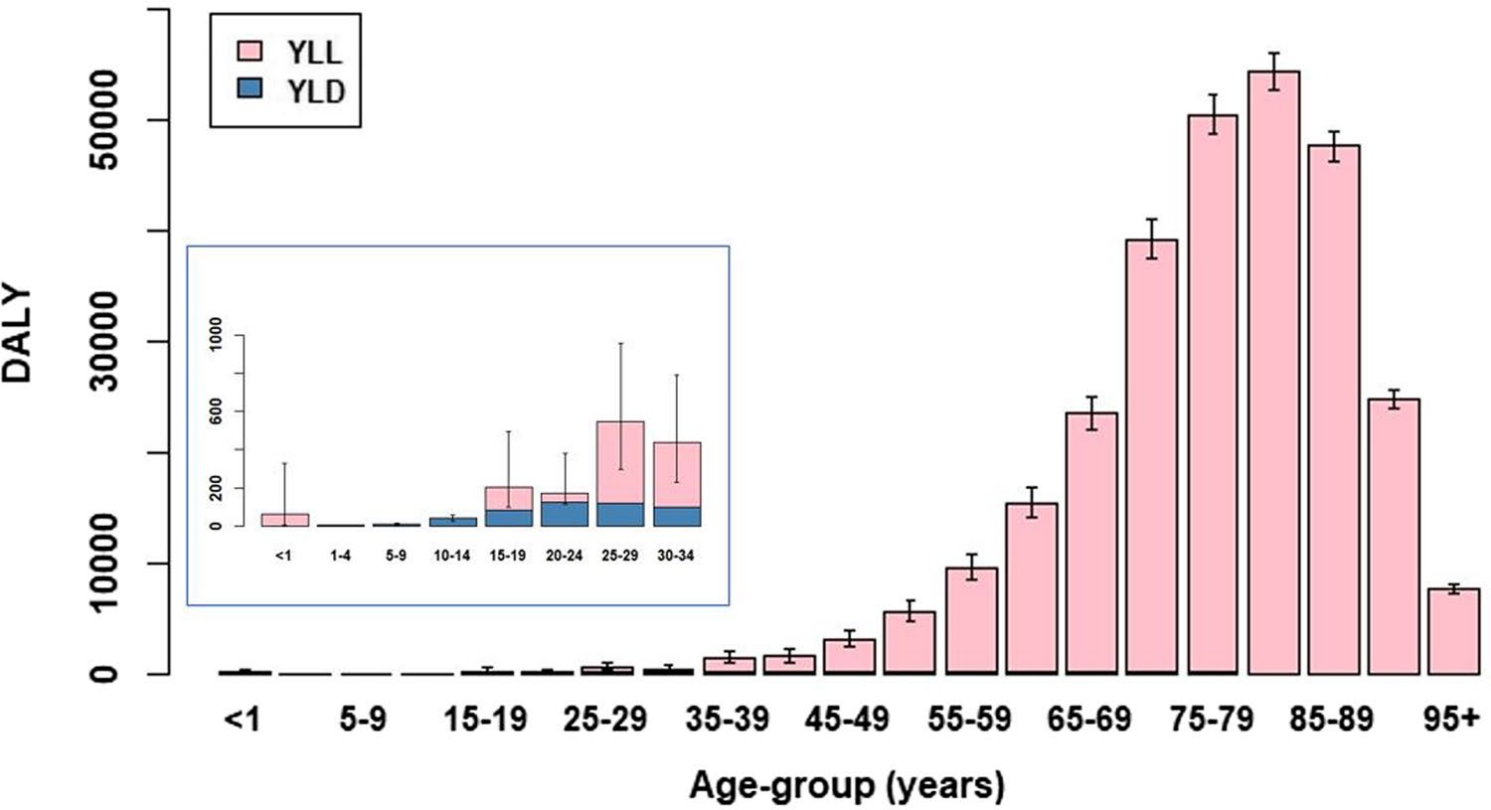
Estimated DALY (split into YLD and YLL) per 5-year age-group with 95% CIs, 27 February through 31 December 2020. Inset zooms in on the age-groups < 1 year to 30–34 years

**Fig. 3:**
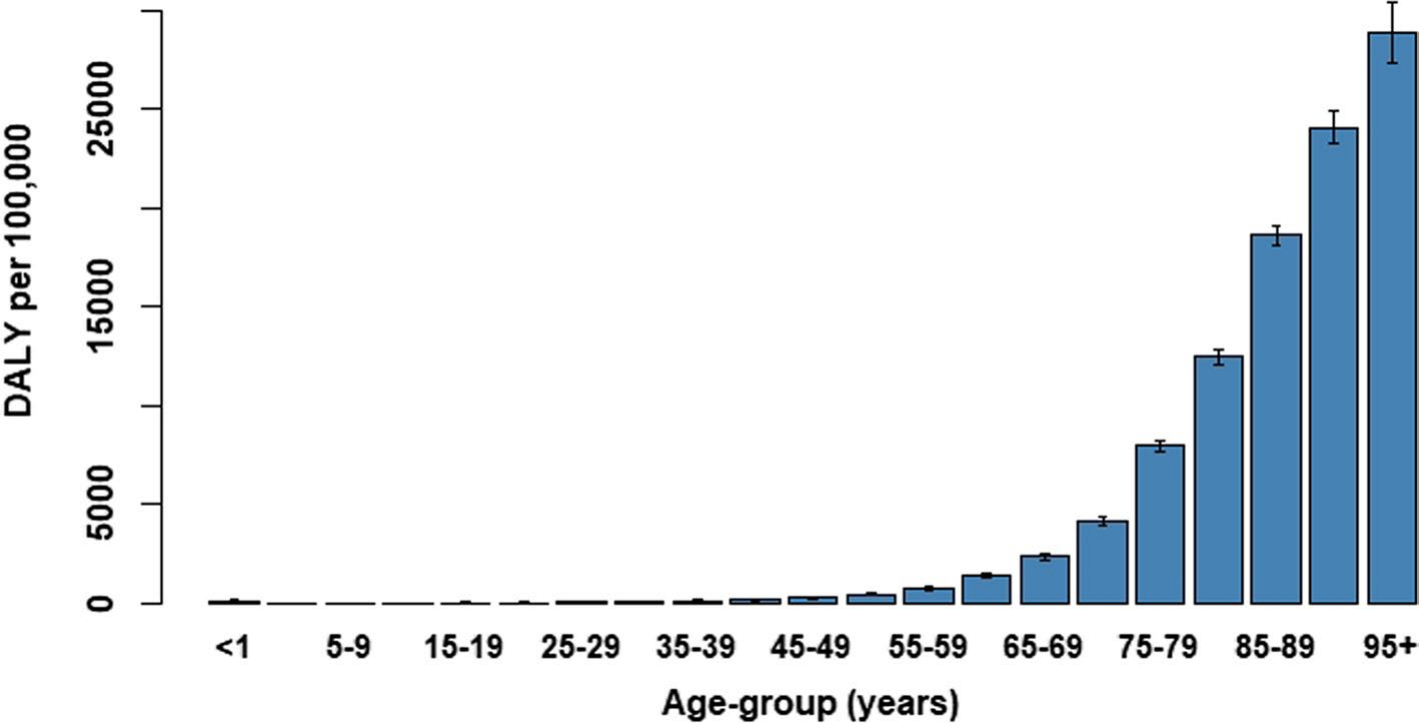
Estimated disease burden per 5-year age-group as DALY per 100,000 population, 27 February through 31 December 2020

Total burden of acute COVID-19 in 2020 was estimated at 286,100 (95% CI: 281,700–290,500) DALY, of which 0.6% was contributed by YLD (Table 2, Fig. 2). A large proportion (36%) of the disease burden among those under 30 years, however, was due to YLD (Fig. 2, inset). DALY per 100,000 population is shown in Fig. 3.

**Table 2:** Estimated cumulative incidence of symptomatic infection, total deaths, DALY, YLD and YLL, analysis period 27 February through 31 December 2020

Health state/indicator	Data or estimate	YLD (95% CI)	YLL (95% CI)	DALY (95% CI)
Mild/moderate*	893,300 (844,600–946,700)	1250 (1180–1320)	–	–
Severe (non-ICU hospital admissions)	28,476	117 (115–119)	–	–
Critical (ICU admissions)	6700	228 (223–234)	–	–
Death	20,173	–	284,500 (280,100–288,900)	–
*Total* *(all health outcomes)*	–	*1600* *(1500–1700)*	*284,500* *(280,100–288,900)*	*286,100* *(281,700–290,500)*

*All symptomatic infection cases (estimated based on the ECDC case definition; see "Methods"), irrespective of whether subsequently admitted to hospital and or ICU

### Estimated cumulative incidence of infection and symptomatic infection

We estimated a cumulative incidence of SARS-CoV-2 infection of 2,571,400 (95% CI: 2,444,900–2,710,700) between 27 February and 31 December, which corresponds to 14.8% (95% CI: 14.0–15.6%) of the total population. The cumulative SI incidence over the same period was estimated at 893,300 (95% CI: 844,600–946,700) (Fig. 1), which is 5.1% of the total population.

The estimated age-aggregated ascertainment of cumulative incidence of infection by the cumulative number of Osiris notified positive cases (*n* = 808,791) over this period was 31% (95% CI: 30–33%). Starting from 1 December 2020, testing was expanded to include asymptomatic persons who had travelled abroad or were identified via contact tracing. As this testing policy change affects only one month of notified case data, an unknown, though likely small, percentage of the total positives in this period were asymptomatic.

### Change in severity over time

In the first analysis period, the DALY/1000 infected persons measure was approximately four times higher than that estimated for the second period (Fig. S5). This reduction in severity over time was due to the estimated total disease burden in the second analysis period (95,000 DALY compared with 191,100 DALY in the first period) decreasing while the cumulative incidence of infection increased (1,698,000 compared with 873,000 infections in the first period).

### DALY stratified by occupation category

**Fig. 4:**
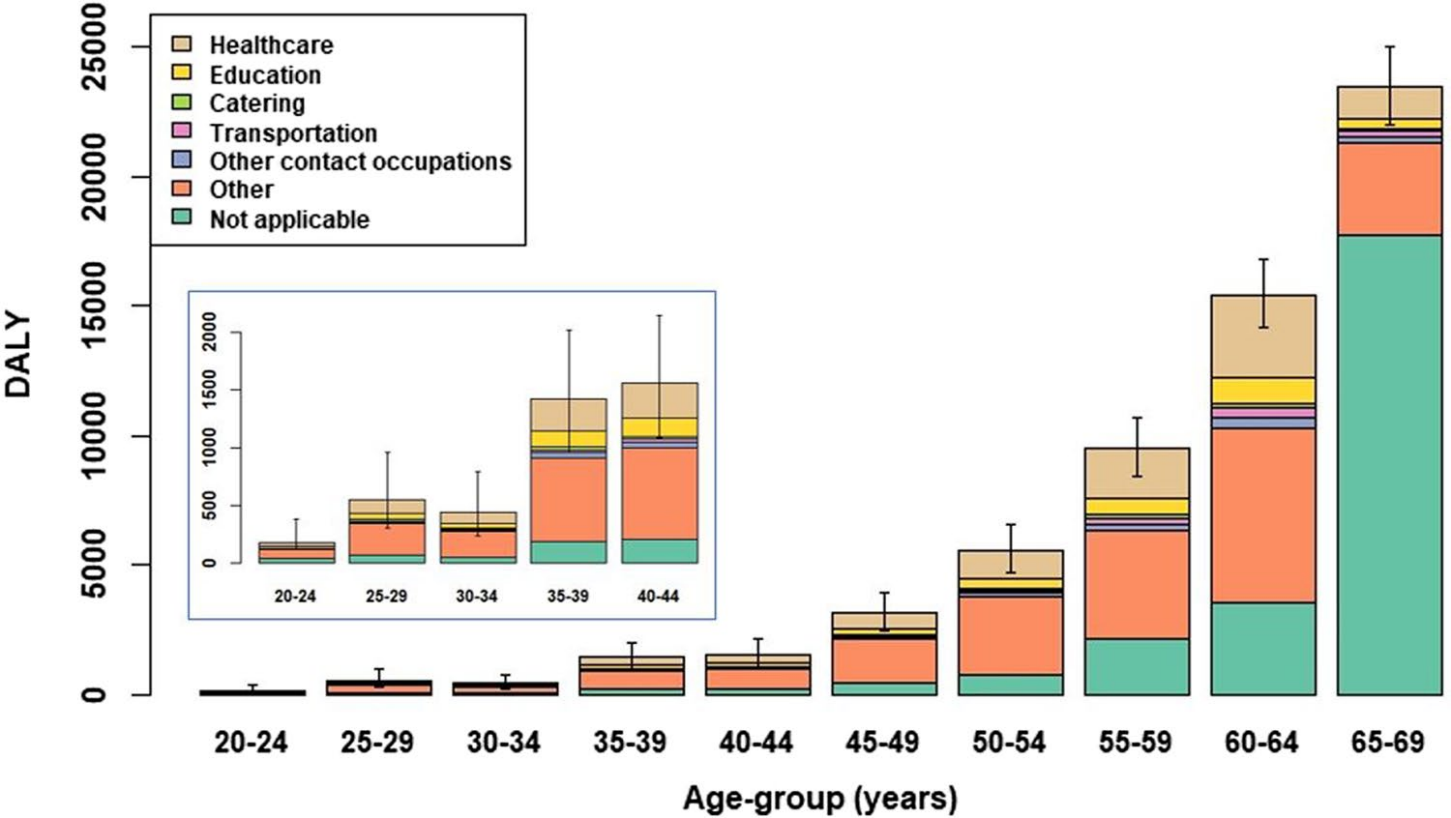
Estimated absolute disease burden (in DALY) per occupation category and 5-year age-group, 27 February through 31 December 2020. Inset zooms in on the age-groups 20–24 years through 40–44 years

**Fig. 5:**
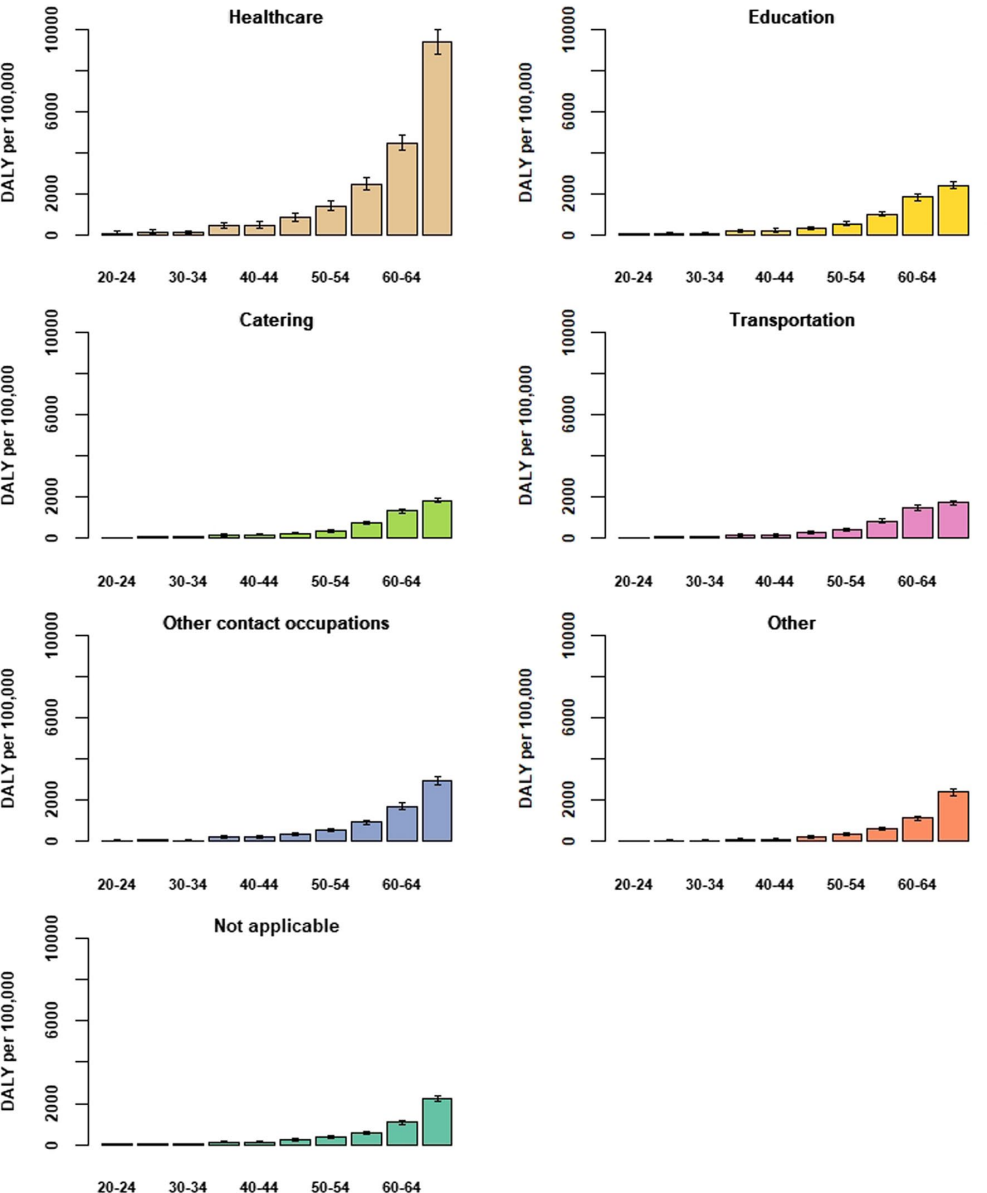
Estimated disease burden per occupation category and 5-year age-group (as DALY per 10,00,000 persons in each category within each age-group), 27 February through 31 December 2020, and shown for the age range 20–69 years only

The absolute burden was greatest for the 'non-working' occupation category (consisting of retired persons, employment seekers and presumably students; Fig. 4), largely because of the much higher mortality burden among older aged retirees. However, when the size of the occupation denominator is taken into account (i.e., the DALY/100,000 measure, aggregating over age), the category *healthcare* appears to bear a disproportionally high relative burden (Fig. S4). The higher relative burden for this category also held when calculated separately per age-group, as the relative disease burden was notably higher than seen for other occupation categories starting from age-group 45–49 years (Fig. 5). The higher relative burden among healthcare workers is attributable to the relatively high estimate of cumulative SI incidence seen across all age-groups for this category (Fig. S5), which presumably reflects a combination of increased workplace exposure and a higher likelihood of being tested.

Supplementary analysis compared the COVID-19 disease burden in individuals receiving long-term institutional health care with community residents in the same age-group (see Supplemental Materials, Table S3 for details). For 2020, the estimated burden for persons aged 80+ years receiving long-term care was 68,700 DALY per 100,000 persons; this was about ten times greater than the estimated 6800 DALY/100,000 persons for individuals aged 80+ years living in the community.

## Discussion

The total disease burden due to acute COVID-19 in the Netherlands was overwhelmingly determined by premature mortality (> 99% of DALY is YLL), in particular from age 35 and up (Fig. 2). The disease burden was unequally distributed over age, with half of the total burden experienced by persons aged 80+ years and with comparatively little burden among persons under 50 years old. The absolute disease burden grew more slowly between our two analysis periods (increasing by 33%), although the estimated cumulative incidence of infection had greatly increased (by 66%). The DALY/1000 infected person measure for the first analysis period (which approximately corresponds to the first wave) was four-fold that estimated for the rest of the year (Supplemental Materials, Fig. S3). This is most likely due to changes in the age-distribution of infected persons (as demonstrated by successive PICO rounds [12]), plus a contribution from improvements in COVID-19 patient prognosis, with as consequence a favourable impact on the risk of severe or fatal outcomes.

Using the relative disease burden measure (DALY/100,000 population), we could compare the per-capita burden between different strata of the population. The (age-aggregated) estimated burden experienced by healthcare workers (approximately 1400 DALY/100,000; Supplemental Materials, Fig. S4) was an order of magnitude lower than the burden experienced by the oldest segment of the population (e.g., 18,500 DALY/100,000 for the age-groups 85–89 years and older; Fig. 3). Although analysis of testing data between June and October 2020 in the Netherlands showed that the occupation sectors catering, public transportation and contact professions had relatively high positivity rates [26], this did not appear to translate to an increased disease burden for these occupations.

### Comparison with the burden of other infectious and chronic diseases

The estimated disease burden of acute COVID-19 for 2020 is approximately 17 times higher than that for a typical influenza season: the average influenza burden was 12,000 DALYs over the five seasons 2015/2016 through 2019/2020 (DALY estimates for these seasons are reported in Table 3.1 of Ref. [4]; we recalculated DALY using GBD-2010 conditional life expectancies [27] to permit comparability with published influenza burden estimates [4, 28]; see Table S2).

DALY for high-burden non-communicable diseases such as coronary heart disease (260,200 DALY) and stroke (228,300 DALY) have been previously estimated for the year 2015 [29]. As these DALY estimates were derived with using national life expectancy tables for the Netherlands, we recalculated COVID-19 DALY using the Netherlands life expectancy values for 2015, arriving at 175,100 DALY (Table S2). This comparison illustrates that the most recent published estimates for the annual burden of coronary heart disease and stroke are higher than the estimated burden of COVID-19 in 2020, when using a common life-table.

### Comparison with estimated COVID-19 burden in other countries

It is important to set the Netherlands estimates into the European and international context. To date DALY estimates using the COVID-19 burden protocol [6] have been produced for Scotland, Germany and Malta for 2020 [31–32]. We could therefore compare the COVID-19 burden in the Netherlands to that estimated for these three countries. The disease burden per 100,00 population in the Netherlands was estimated at 1640 DALY (95% CI: 1620–1670). Table 3 shows how this figure compares with other countries' estimates using similar approaches. We note that interpretation of YLD differences between countries should recognise differences in testing policies and behaviour, and for interpreting YLL differences one must consider potential differences in COVID-19 death under-reporting rates. Among the four countries, Scotland has reported the highest per capita COVID-19 burden – this estimate includes burden due to post-acute consequences – and Germany reported the lowest per-capita burden. The per capita burden estimate for the Netherlands is 4.5 times greater than for Germany, in part due to differences in normative life expectancies. When YLL for Germany is also calculated using the GBD-2019 tables, YLL is 1.5 times higher (A. Wengler, pers. comm.), increasing the relative disease burden from 368 to 542 DALY/100,000. Although GBD-2019 life expectancy values were used for both the Scotland and Netherlands estimates, YLL/100,000 is 13% lower in the Netherlands as compared to Scotland, despite the fact that the number of COVID-19 fatal cases per 100,000 was quite similar in the two countries. This suggests that the average age at death is younger in Scotland. In summary, mortality appears to be the main driver of these between-country differences. Given that the Netherlands, Scotland and Germany have broadly similar demographics, differences in the DALY per 100,000 measure reflect relative success in protecting the elderly and vulnerable segment of the population from SARS-CoV-2 infection.

**Table 3:** Between-country comparison of COVID-19 disease burden

Country and analysis period	Estimation of total symptomatic infected	Life expectancy table	Mortality due to COVID	Include post-acute	DALY/100,000	% YLD
Netherlands [2020]	Yes^a^ (evidence synthesis)	GBD-2019	Statistics Netherlands registered (confirmed + suspected)	No	1640 (1620–1670)	1%
Scotland [2020]	Yes (SEIR modelling)	GBD-2019	Death registry (confirmed only or confirmed + suspected)	Yes, limited	1770–1980	2%
Germany [2020]	No (notified positives only)	Germany 2016/2018	Deaths among notified cases	No	368	0.7%
Malta [7 Mar 2020—31 Mar 2021]	Yes (notified positives adjusted for underascert.)	GBD-2019	Daily COVID-19 bulletins issued by Malta Ministry of Health	Yes, limited	1086^b^	5%

^a^For the Netherlands, 'total symptomatic infected' is derived using the ECDC case definition (see "Methods")

^b^Calculated based on the reported estimate of 5478 DALY, in population size of 505,200 (estimate for 2019; World Bank)

Our estimates covered the calendar year 2020, to facilitate comparison with the COVID-19 burden in other countries, and with the routinely reported burden of other infectious diseases in the Netherlands. However, although the peak of the second wave (based on notification data) was in December 2020, the end of this wave occurred around the end of January [1], and mortality among persons infected during the last part of the second wave would be observed until approximately the end of February. Therefore, based on published mortality figures [33] we estimated the additional YLL until the end of the second wave (in January 2021), and also when including the associated fatal cases (1 January until 28 February 2021). These were 56,200 (95% CI: 53,800 -58,600) and 91,700 (95% CI: 88,700–94,900) DALY in January 2021 and January/February 2021, respectively.

As we have shown, for meaningful across-country and between-disease DALY comparisons, the same life-table must be used in the calculation of YLL. Furthermore, we have chosen to use the aspirational life expectancy approach, for which age at death is the only relevant factor. When DALY are used to inform public health decision-making, it is important that certain subpopulations (whether defined by a higher prevalence of comorbidities, lifestyle risk factors, or degree of socio-economic deprivation) are not disadvantaged for receipt of prevention or treatment interventions because they have a lower expected remaining life expectancy than other subpopulations [34].

Strengths of this study include making use of all relevant data sources to estimate the disease burden, and the adoption of a developed protocol for estimation of the COVID-19 disease burden. We have identified the following limitations. First, the total disease burden for the period until 31 December 2020 presented here is known to underestimate the true burden because health outcomes following the resolution of acute infection (i.e., sequelae, often known as 'long COVID') have not yet been included. Current knowledge regarding post-COVID-19 syndrome is that it can be described as constellations of symptoms affecting different physiological systems that can vary in severity and duration [35], but early estimates indicate its contribution to the total disease burden is on the order of 1–3% [30, 32]. As more data on progression risk, severity, and duration come available [36, 37], the current estimates can be revised to include the burden attributable to the long-term sequelae of SARS-CoV-2 infection.

Second, the estimated relative disease burden per occupation category must be interpreted with caution as there were limitations to the data sources and the consequent possible analyses. For a given occupation category, the relative disease burden was estimated for the entire analysis period and is not necessarily indicative of the recent burden; for instance, widespread availability of personal protective equipment and other risk-reducing measures may mean that the proportion of burden experienced by healthcare workers over the last half of the year was likely much reduced. Related to this point, DALY per occupation category was derived using the distribution of notified cases over the entire year, thus aggregating together periods of relatively 'open' society with periods in which strict measures were in place. The procedure also combined periods in which there was non-universal access to testing (i.e., before 1 June 2020, priority was given to severe/hospitalised cases) and/or priority testing for certain occupations, such as healthcare workers and the education sector, and so the distribution of occupation categories among notified cases is influenced by access to testing; with periods in which there were minimal public health restrictions in place, with (near) universal access to testing.

When strict measures were in place, some occupations could not be practiced; for others, contact patterns and ensuing transmission risk in the workplace setting might be quite different. As an example, the proportion in category 'education' will not be fully representative of the normal term-time situation with in-person teaching, due to the (partial) continuation of online teaching after 1 June 2020, and the school holiday period. A further assumption is that the occupation provided in a notified case's Osiris record applied throughout the analysis period (i.e., person was not (temporarily) inactive in their occupation, and did not become unemployed). In addition, our approach did not take into account possible variation in the risk of severe disease and/or mortality by occupation, because the occupation distribution (per age-group) is applied to the total burden (for that age-group). For instance, if (conditioning on age) healthcare workers have better underlying health and therefore better prognosis [38], or are more skilled in risk perception and personal health management, compared with other occupations, then both the absolute and relative disease burden will have been overestimated for this occupation category.

Third, our estimate of the cumulative SI incidence depends on the age-specific attributable risk derived symptomatic proportion. This method estimates the proportion of infections for which symptoms can uniquely be attributed to SARS-CoV-2 infection and as such represents an lower bound for the true proportion; mild symptoms that also occur with other afflictions (e.g., common cold, hay fever) are thus discounted. This would lead to an underestimation of YLD, but would have a very small impact on DALY due to the overwhelming contribution of YLL. Finally, disability duration post-hospital/ICU discharge (time until recovery) was not estimated or included, which would also lead to an underestimation of YLD, and improvements in treatment over time, potentially leading to short hospital stay, were not considered.

We have presented the real-world disease burden estimates, i.e., as derived from infections that occurred during a period in which (partial) lockdown measures were in place more often than not. We did not attempt to calculate counterfactuals – what would the disease burden have been if no measures were imposed? How much could the burden have been reduced if stricter measures were taken, or at earlier stages of the epidemic? Although such alternative scenarios are potentially useful for evaluation and future planning, for a number of infectious agents – whether the cause of large historical outbreaks or endemic situations—widespread population interventions were not been considered feasible, and so the best use of disease burden estimates is to inform planning and prioritisation based on the data generated by real-world situations.

The primary focus of this work is the morbidity and mortality *directly* caused by SARS-CoV-2. The impact of health care displaced or delayed by COVID-19 patients (i.e., the indirect impact of the pandemic) has been calculated to be on the order of 34,000 to 50,000 lost healthy-life years (QALY) up to 31 August 2020 [39]. In addition, the imposition of non-pharmaceutical control measures such as social distancing and lockdown has almost certainly had a toll on mental health, the burden of which still needs to be estimated.

## Conclusion

Estimates of the acute COVID-19 disease burden are important for establishing the direct impact of the SARS-CoV-2 pandemic on population health, for guiding the deployment of preventive measures, such as vaccination, and for informing economic evaluations. When examined in the context of other high-burden infectious and non-communicable diseases, it is clear that COVID-19 was responsible for a very high burden of disease in the Netherlands in 2020, despite extensive control measures. Furthermore, age was the main source of variation in estimated disease burden, driven by the sharply skewed age-specific mortality risk.

## Supplementary Information

Below is the link to the electronic supplementary material.

Supplementary file1 (DOCX 453 KB)

## Data Availability

Several of the datasets used in the current study are available in the RIVM Open Data Repository (https://data.rivm.nl/covid-19/). Other data are accessible to researchers upon reasonable request to the corresponding author.

## Abbreviations

CI: Confidence interval
COVID-19: Disease caused by the SARS-CoV-2 (2019) virus
DALY: Disability adjusted life years
ECDC: European centre for disease prevention and control
GBD: Global burden of disease study
ICU: Intensive care unit
IgG: Immunoglobulin G
NICE: National intensive care evaluations
ONS: Office of national statistics
PICO: PIENTER corona study
QALY: Quality adjusted life years
SARS-CoV-2: Severe acute respiratory syndrome coronavirus 2019
SEIR: Suspectible-exposed-infectious-recovered
SI: Symptomatic infection
YLD: Years lived with disability
YLL: Years of life lost
