# Supplementary material for: The estimated disease burden of acute COVID-19 in the Netherlands in 2020, in disability-adjusted life-years
Source: Eur J Epidemiol. 2022 Aug 11;37(10):1035–47. doi: 10.1007/s10654-022-00895-0 (PMC9366822; doi:10.1007/s10654-022-00895-0)

**SUPPLEMENTARY MATERIALS**

*To accompany the manuscript:*

The estimated disease burden of acute COVID-19 in the Netherlands in 2020, in
disability-adjusted life-years

Scott A. McDonald, Giske R. Lagerweij, Pieter de Boer, Hester E. de Melker, Roan Pijnacker, Lapo Mughini Gras, Mirjam E. Kretzschmar, Gerco den Hartog, Arianne B. van Gageldonk-Lafeber, RIVM COVID-19 surveillance and epidemiology team, Susan van den Hof, Jacco Wallinga

Corresponding author:

Scott A. McDonald

Center for Infectious Disease Control, National Institute for Public Health and the Environment (RIVM), Bilthoven, the Netherlands

E-mail: scott.mcdonald@rivm.nl

**Computation of YLD, YLL and DALY**

There a several good sources describing the calculation of disability-adjusted life years (DALY) (see references, below), and at least one freely available R package for calculation (the *DALY Calculator*; see reference below).

**Estimation of years lived with disability (YLD)**

For each of *N* health states in the clinical pathway progression diagram (Fig. S2), there are three parameters which require data: incidence, duration, and disability (or severity). YLD are calculated by summing the product of the incident number of persons within the health state, duration in the health state, and the disability weight, across all health states:

YLD $= Incidence \times Duration \times Disability weight$

$$Total YLD= \sum_{H=1}^{N} {YLD}_{H}= \sum_{H=1}^{N} {Incidence}_{H}\times{Duration}_{H}\times{Disability weight}_{H}$$

where *H* = health state and *N* = total number of health states.

**Estimation of years lost to premature mortality (YLL)**

When estimating YLL, two parameters are required: the number of deaths and the conditional life expectancy, both of which are typically defined per (multi-year) age-group. YLL is estimated as:

$$YLL= Number of deaths \times Life expectancy$$

$$Total YLL=\sum_{A=1}^{M} {YLL}_{A}=\sum_{A=1}^{M} {Number of deaths}_{A}\times{Life expectancy}_{A}$$

where A = age-group, e.g. 1-year or 5-year groups, M = total number of age-groups. Age-group conditional life expectancy can be defined from life tables (e.g. national, regional, or aspirational).

**Estimation of DALY**

$$DALY= YLD+ YLL$$

$$Total DALY=\sum_{A=1}^{M} {DALY}_{A}=\sum_{A=1}^{M} {YLD}_{A}+ {YLL}_{A}$$

**References**

Devleesschauwer B, McDonald S, Haagsma J, Praet N, Havelaar A, Speybroeck N. DALY: The DALY Calculator - Graphical User Interface for probabilistic DALY calculation in R. 2016. <http://daly.cbra.be/>

Devleesschauwer B, Havelaar AH, Maertens de Noordhout C, Haagsma JA, Praet N, Dorny P, Duchateau L, Torgerson PR, Van Oyen H, Speybroeck N. DALY calculation in practice: a stepwise approach. *Int J Public Health.* 2014; 59:571-574.

Mathers CD, Vos T, Lopez AD, Salomon J, Ezzati M. National burden of disease studies: a practical guide. 2001. Geneva: World Health Organization.

**Table S1.** Definition of occupation categories and proposed set of denominator occupations from Statistics Netherlands (CBS).

| **Occupation category** | **Occupation label(s) in Osiris (translated from Dutch)** | **CBS occupation category(s) for population denominator (translated from Dutch)** |
| --- | --- | --- |
| Healthcare | (Health)care | 1011 Doctors  1012 Specialised nurses  1033 Nurses  1034 Medical practice assistants  1051 Carers |
| Education | Education and daycare | 0111 Higher education lecturers and professors  0112 Teachers (vocational training)  0113 Teachers (secondary school, general  subjects)  0114 Teachers (primary school)  0115 Education specialists and other teachers  0121 Sports instructors  0131 Daycare staff and education assistants |
| Catering | Catering sector employee | 1112 Cooks  1113 Waiting staff and bar personnel  1122 Kitchen porters |
| Transportation | Transportation | 1211 Seaman and pilots  1212 Chauffeurs, taxi, and delivery drivers  1213 Bus and tram drivers  1214 Lorry drivers |
| Other contact professions | Other contact professions  Sex work | 1013 Physiotherapists  1035 Medical specialists  1114 Barbers, hairdressers and estheticians  1116 Providers of other personal services  (including driving instructors, sex workers) |
| Other | Clinical laboratory  Agriculture  Other sector  Work with animals or animal  products  Landscape gardening  Waste processing  Cleaning sector  Outside of the Netherlands | *Denominator calculated as [age-group-specific] 'total working population size' minus sum of above categories* |
| Not applicable | Not applicable (children, pensioners, job-seekers) | *Denominator calculated as [age-group-specific] national population size minus sum of all above categories* |

**Table S2.** Estimated DALY, YLD and YLL due to COVID-19 (analysis period 27 February through 31 December 2020), comparing estimates derived using GBD-2019, GBD-2010, and Dutch (for the year 2015) conditional life expectancy values.

| Normative life expectancy values | YLD (95% CI) | YLL (95% CI) | DALY (95% CI) | DALY per 100,000 (95% CI) |  | DALY (95% CI) |
| --- | --- | --- | --- | --- | --- | --- |
| GBD-2019  (main analysis) | 1600  (1500-1700) | 284,500  (280,100-288,900) | 286,100 (281,700-290,500) | 1640 (1620-1670) |  |  |
| GBD-2010 | 1600  (1500-1700) | 205.400 (202,000-208,800) | 207,000  (203,600-210,400) | 1190 (1170-1210) |  |  |
| Netherlands 2015 | 1600 (1500-1700) | 173,500 (170.500-176,400) | 175,100  (172,100-178,000) | 1010 (990-1020) |  |  |

**Table S3.** Derivation of mortality burden among elderly persons receiving institutional care.

|  |  | Institutional care recipients  in the Netherlands | | Not institutional care  recipients | |
| --- | --- | --- | --- | --- | --- |
| 5-year age-group | Life expectancy from GBD-2019 (years)^[1]^ | COVID-19 deaths^[2]^ | Years of life lost (YLL) | COVID-19 deaths^[2]^ | Years of life lost (YLL) |
| 80-84 | 13.24 | 2235 | 29590 | 1780 | 23570 |
| 85-89 | 9.99 | 3180 | 31770 | 1585 | 15830 |
| 90-94 | 7.62 | 2630 | 20040 | 800 | 6090 |
| 95+ | 5.92 | 1280 | 7580 | 205 | 1210 |
| **80+** | -- | **9325** | **88970** | **4370** | **46710** |

Given the population sizes of 129,575 [3] and 692,513 [4] for persons aged 80+ in 2020 in long-term institutional care (i.e., residents of nursing homes, elderly or disabled care facilities, or receiving full-time care at home) and not in institutional care, respectively, YLL was estimated at 68,700 and 6,800 per 100,000 population, respectively.

**Sources**

1. Global Burden of Disease Collaborative Network. Global Burden of Disease Study 2017 (GBD 2019) Reference Life Table. Seattle, United States: Institute for Health Metrics and Evaluation (IHME), 2021. http://ghdx.healthdata.org/gbd-2019.

2. *Data on the total number of COVID-19 deaths among persons receiving and not receiving long-term institutional care in 2020, for those aged 80 years and older (n=9325; 4370, respectively); data available per age-group to calculate the age-group specific YLL among each group.*

Statistics Netherlands. Overledenen per maand naar Wlz-gebruik, leeftijd en doodsoorzaak COVID-19, 2020 en 2021 (tot en met november). [Monthly deaths according to use of compulsory contribution long-term care, age, and COVID-19 cause of death, 2020 and 2021 (until November)]. 25 March 2022. Available from: https://www.monitorlangdurigezorg.nl/publicaties/maatwerk-publicaties/2022/03/25/overledenen-per-maand-naar-wlz-gebruik--leeftijd-en-doodsoorzaak-covid-19-2020--2021-tm-november. Accessed 28 March 2022.

*3. The population size of individuals receiving long-term institutional care includes those individuals receiving care in nursing homes, and elderly or disabled care facilities, and individuals receiving care at home.*

Statistics Netherlands. Monitor Long-term Care; use of compulsory contribution long-term care by care type, care package, and region. Available from: <https://mlzopendata.cbs.nl/#/MLZ/nl/dataset/40055NED/table?dl=63D29>. Accessed 15 March 2022.

4. Statistics Netherlands. Population; key figures. CBS; 13 Oct 2021. Available from: https://opendata.cbs.nl/#/CBS/en/dataset/37296eng/table?ts=1646313474977. Accessed 3 March 2022.

**Fig. S1**. Total weekly deaths among SARS-CoV-2 positive cases from the Osiris case notification system, plotted over the full period (27 February through 31 December 2020). The vertical line marks the division into the two analysis periods (estimated DALY in each plotted in Fig. S4).

**
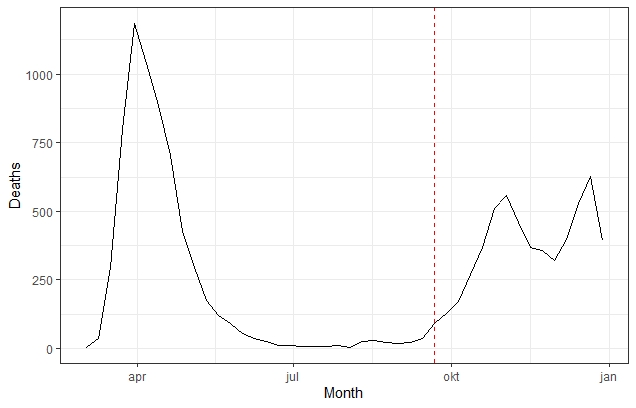
**

**Fig. S2**. Clinical pathway progression diagram for COVID-19.


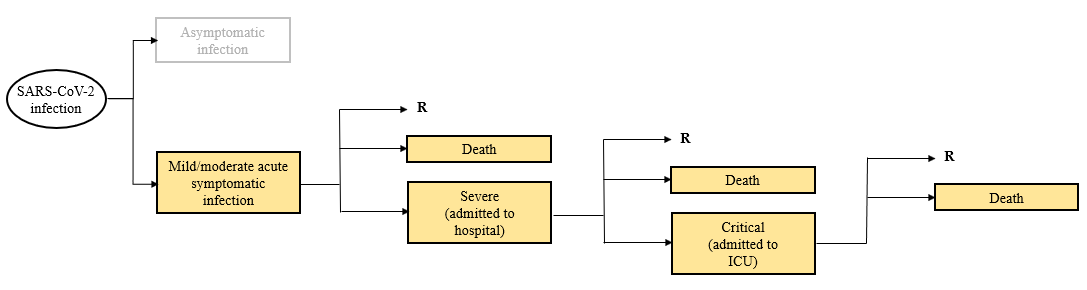


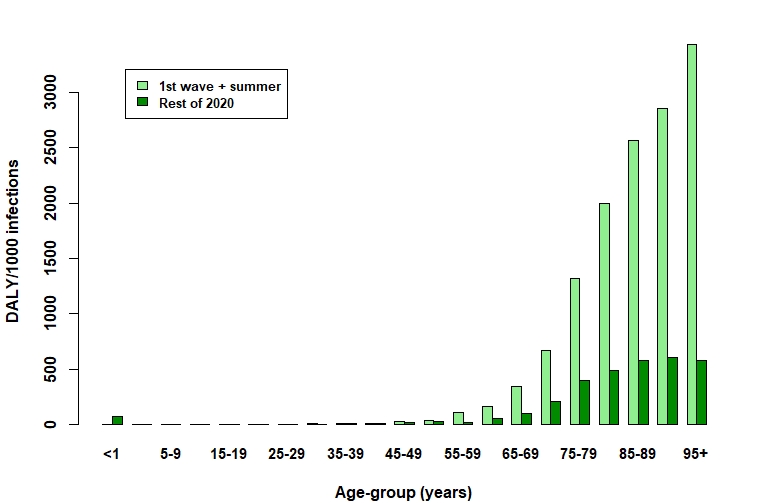
**Fig. S3**. Estimated DALY per 1000 SARS-CoV-2 infected persons, stratified by 5-year age-group and analysis period (i.e., first wave + summer period compared to the rest of 2020).

**Fig. S4.** Estimated disease burden per occupation category (as DALY per 100,0000 persons in each category, aggregating over age and restricted to the age range 20-69 years only), 27 February through 31 December 2020.


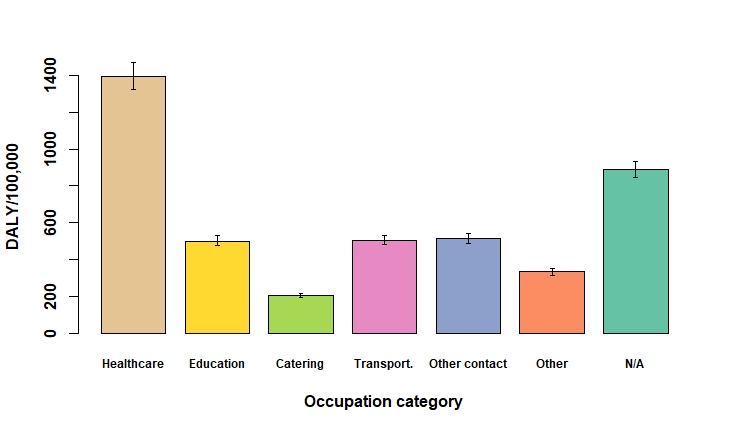


**Fig. S5**. Estimated cumulative incidence (per 100,000) of mild/moderate cases per occupation category and 5-year age-group (as the estimated total number of patients per 100,000 persons in each category within each age-group), 27 February through 31 December 2020 and shown for the age range 20-69 years only.


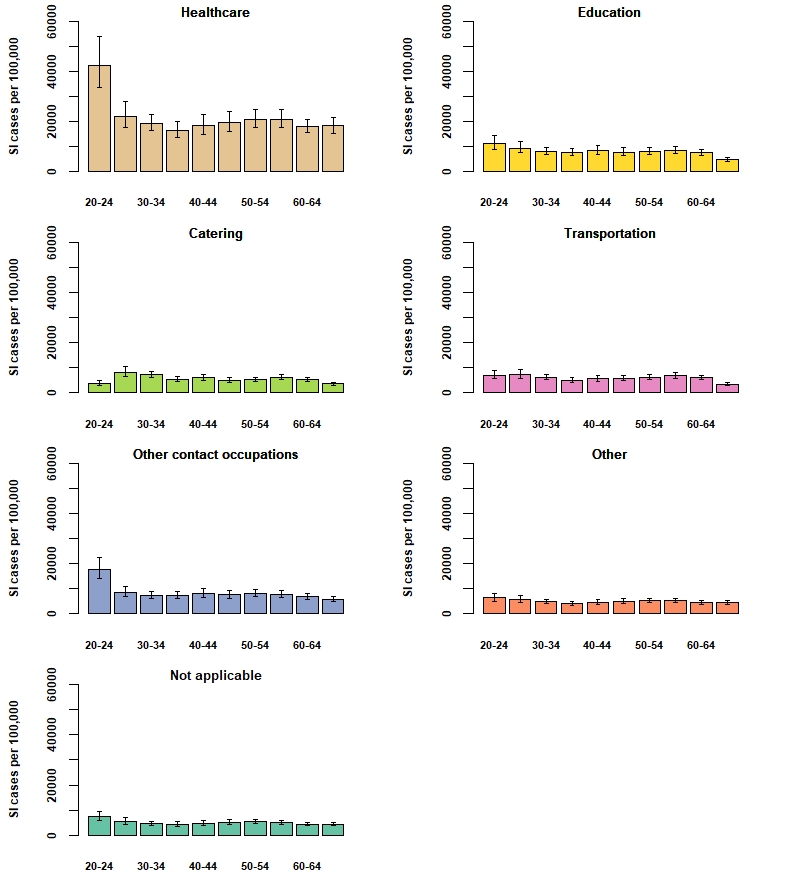

Supplement: Supplementary file 1 — Supplementary file1 (DOCX 453 KB) [file 10654_2022_895_MOESM1_ESM.docx]
